# The Largest Hospitals

**Published:** 1911-03-11

**Authors:** S. Holland

**Affiliations:** Kneesworth Hall, Royston, Herts.


### Editor's Letter-Box.

### THE LARGEST HOSPITALS.

*To the Editor of* The Hospital.

Sir,—"R.F.P.—You are quite wrong. The London Hospital is by no means the largest institution of its kind in the world. The largest hospitals are the Virchow at Berlin and the Maggiore at Milan. The London has the distinction of being the largest voluntary hospital in the British Empire."

I am afraid this is not accurate. I rather think that the Edinburgh Infirmary has a few more. In Burdett's "Hospitals, etc.," the London has 922 with 799 daily occupied. Edinburgh has 926, 836 daily occupied. I wonder if this 836 is accurate. It may be if they do not reserve beds for erysipelas, diphtheria, and so on. I have always been careful to claim for the London only "the largest Hospital in England."—Yours faithfully,

(Sgd.) S. Holland.

Kneesworth Hall, Royston, Herts.

[Mr. Holland raises an interesting point on which we should like the opinion of the Edinburgh authorities.—Ed., The Hospital.]

